# Comparison of Levitation Properties between Bulk High-Temperature Superconductor Blocks and High-Temperature Superconductor Tape Stacks Prepared from Commercial Coated Conductors

**DOI:** 10.3390/ma17184516

**Published:** 2024-09-14

**Authors:** Anke Kirchner, Tilo Espenhahn, Sebastian Klug, Kornelius Nielsch, Ruben Hühne

**Affiliations:** 1 Leibniz Institute for Solid State and Materials Research, Helmholtzstr. 20, 01069 Dresden, Germany; a.kirchner@ifw-dresden.de (A.K.); t.espenhahn@ifw-dresden.de (T.E.); s.klug@hzdr.de (S.K.); k.nielsch@ifw-dresden.de (K.N.); 2 Institute for Materials, TUD Dresden University of Technology, 01062 Dresden, Germany

**Keywords:** high-temperature superconductors, REBCO bulk material, coated conductors, tape stacks, levitation force

## Abstract

Bulk high-temperature superconductors (HTSs) such as *RE*Ba_2_Cu_3_O_7−x_ (*RE*BCO, *RE* = Y, Gd) are commonly used in rotationally symmetric superconducting magnetic bearings. However, such bulks have several disadvantages such as brittleness, limited availability and high costs due to the time-consuming and energy-intensive fabrication process. Alternatively, tape stacks of HTS-coated conductors might be used for these devices promising an improved bearing efficiency due to a simplification of manufacturing processes for the required shapes, higher mechanical strength, improved thermal performance, higher availability and therefore potentially reduced costs. Hence, tape stacks with a base area of (12 × 12) mm^2^ and a height of up to 12 mm were prepared and compared to commercial bulks of the same size. The trapped field measurements at 77 K showed slightly higher values for the tape stacks if compared to bulks with the same size. Afterwards, the maximum levitation forces in zero field (ZFC) and field cooling (FC) modes were measured while approaching a permanent magnet, which allows the stiffness in the vertical and lateral directions to be determined. Similar levitation forces were measured in the vertical direction for bulk samples and tape stacks in ZFC and FC modes, whereas the lateral forces were almost zero for stacks with the *RE*BCO films parallel to the magnet. A 90° rotation of the tape stacks with respect to the magnet results in the opposite behavior, i.e., a high lateral but negligible vertical stiffness. This anisotropy originates from the arrangement of decoupled superconducting layers in the tape stacks. Therefore, a combination of stacks with vertical and lateral alignment is required for stable levitation in a bearing.

## 1. Introduction

High-temperature superconductors (HTSs) offer appealing advantages for electric power applications if compared with conventional materials. Examples are a significant higher energy efficiency and higher energy densities in superconducting generators, motors, transformers and cables [1,2]. Additionally, novel grid technologies were developed as superconducting current limiters or superconducting magnetic energy storage devices showing properties not accessible by conventional systems. Some of these devices include superconducting magnetic bearings (SMBs), where typically the HTS compound *RE*Ba_2_Cu_3_O_7−x_ (*RE*BCO, *RE* = rare earth, e.g., Y, Gd) is used as bulk material. Examples are rotationally symmetric SMBs applied in motors or flywheels [3,4,5,6,7]. The advantage of these SMBs is the significantly reduced friction, which leads to reduced operational losses. Such bearings are also studied for new applications, for example, in textile machines [8,9,10]. Here, higher maximum rotational speeds than with the conventional system were realized as shown for an SMB-based twist element in ring spinning machines.

A rotational SMB usually consists of a permanent magnet ring, which is combined with an arrangement of commercially available *RE*BCO bulk material [5]. The magnetic field profile of the permanent magnet ring is fixed inside the superconducting material during the field cooling process, resulting in the stable levitation of the ring above the superconductor. However, the application of bulks in SMBs has several disadvantages such as restricted flexibility due to the brittleness of the material and the limited availability and high costs due to the time-consuming and energy-intensive fabrication process [11,12].

As alternative, tape stacks of HTS-coated conductors might be used for these devices promising an improved bearing efficiency due to a simplification of manufacturing processes for the required shapes, higher mechanical strength, improved thermal performance, higher availability and therefore potentially reduced costs [13,14]. The potential of such coated conductor stacks for bearing applications is demonstrated, among other factors, by the fact that with a superconductor volume fraction of only 1.5%, half of the load capacity is reached in fields oriented perpendicular to the tape surface, if compared to an SMB with bulk material [15]. On the other hand, significantly lower bearing forces or lateral guiding forces are present if the tape planes are aligned parallel to the magnetic field since the high resistance between the neighboring *RE*BCO layers prevents superconducting ring currents for this orientation [16]. Therefore, the intrinsic anisotropy of the conductor material itself is a major challenge for the application of tape stacks in levitation systems.

HTS-coated conductor tapes are nowadays commercially available from a number of companies. They are fabricated by various deposition techniques and were mainly optimized for applications in cables or coils, for which a high current-carrying capability in magnetic fields is important [17]. This is typically achieved by the incorporation of dedicated artificial pinning centers, which are mostly optimized for current transport in cables. These pinning centers are of high importance when using coated conductors as tape stacks in bearings as they fix the magnetic field configuration inside the superconductor. During operation, external forces might influence the rotating permanent magnet ring due to vibrations or disturbances leading to a vertical or lateral displacement, which is equivalent to a change in the magnetic field profile inside the superconducting material. In such cases, high restoring forces in the vertical (*F*_z_) and lateral (*F*_y_) directions are beneficial and are provided by efficient flux pinning centers inside the *RE*BCO superconductor. In general, the behavior of such a bearing can be described by the lateral and vertical stiffnesses, which are dependent on the critical current density *j*_c_. For bulk materials, as for tape stacks, this value might be estimated from trapped field measurements, where the field gradient *dB*/*dx* is equivalent to the *J*_c_ value according to the Bean model [18].

In recent years, a number of basic studies have been performed on the application of HTSC tape stacks for levitation [12,13,15,16,19,20,21,22]. It was realized that application-relevant properties such as stiffness and trapped fields depend on various characteristics of the tape stacks, such as the critical current density of the tapes and the number of layers used [20] as well as the arrangement of the layers in the stack itself [14]. The goal of our study is to compare tape stacks with commercial bulk materials with the same geometry in order to check whether such stacks can simply replace the YBCO blocks used in existing SMB systems. To do this, we focus on an arrangement, which is equivalent to a setup used in an SMB for ring spinning, where an axially magnetized permanent magnet ring levitates above concentrically arranged bulk superconductors [8]. It should be noted that the magnet can move freely in the vertical and lateral directions; i.e., we need to ensure a high stiffness against movements in both directions to realize a stable levitation state. To quantify the behavior in more detail, we study small displacements of a few millimeters only which might appear during the operation of such a bearing due to external forces or excitation of the bearing by machine vibrations in a resonance case [9,23]. Furthermore, our emphasis is to investigate the influence of the stack height on the levitation properties, which allows the amount of material used to be optimized. Finally, we check whether an arrangement of stacks with different orientations can solve the challenge of the anisotropic behavior of such stacks.

## 2. Experiment

### 2.1. Sample Preparation

**Figure 1:**
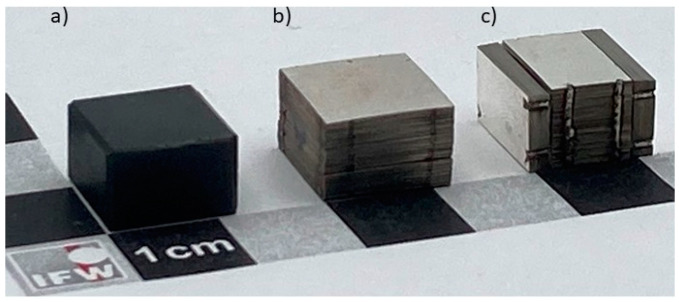
Images of selected samples: (**a**) YBCO bulk; (**b**) tape stack prepared from GdBCO-coated conductors; (**c**) combination of stacks with different orientations to the magnetic field. The dimensions of all samples shown here are 12 mm × 12 mm × 8 mm.

Commercial YBa_2_CuO_7−x_ (YBCO) bulks from CAN Superconductors (Říčany, Czech Republic) with an area of (12 × 12) mm^2^ and sample heights of 2.5 mm, 5 mm, 8 mm and 12 mm were used as reference samples for our study. The levitation properties of these bulk samples were compared to tape stacks prepared from commercially available *RE*BCO tapes produced by Theva Dünnschichttechnik GmbH (Ismaning, Germany). The 12 mm wide HTS tape exhibits a layer architecture with a 50 µm Hastelloy substrate, an about 2 µm thick MgO buffer, an about 3 µm thick GdBa_2_Cu_3_O_7−x_ (GdBCO) layer and a surrounding silver metallization. The average critical current for the tape given by the company was 770 A at 77 K. The tape was cut into (12 × 12) mm^2^ pieces, which were stacked on top of each other without changing the orientation of the pieces and joined laterally by two laser welding seams on each side. The number of tapes in the stacks was chosen to achieve a stack height equivalent to the bulk samples, as shown in Figure 1. More specifically, 45, 90, 145 and 215 tapes were used for the 2.5 mm, 5 mm, 8 mm and 12 mm thick stacks, respectively. In addition, a combined stack with an overall surface area of (12 × 12) mm^2^ and a height of 8 mm was produced. This sample consists of a middle stack with a base area of (12 × 8) mm^2^ and a height of 8 mm and two stacks arranged perpendicular to the middle stack with a base area of (12 × 8) mm^2^ and a height of 2 mm; see Figure 1c.

### 2.2. Characterization

At first, the trapped field *B*_z_ was studied for all samples using scanning Hall probe magnetometry. Two cases were considered: On the one hand, the measurement was performed after an external magnetization using a parallel field of 3 Tesla produced by an open-bore Oxford magnet. This field is large enough to saturate the samples at 77 K. For a second set of measurements, the magnetic field was provided during cooldown in liquid nitrogen by a standard NdFeB magnet with a surface field of about 0.35 T. This arrangement resembles the typical field cooling conditions in an SMB. For both cases, the trapped field was measured at 77 K via a plane scan with a Hall probe positioned 0.5 mm above the sample surface.

**Figure 2:**
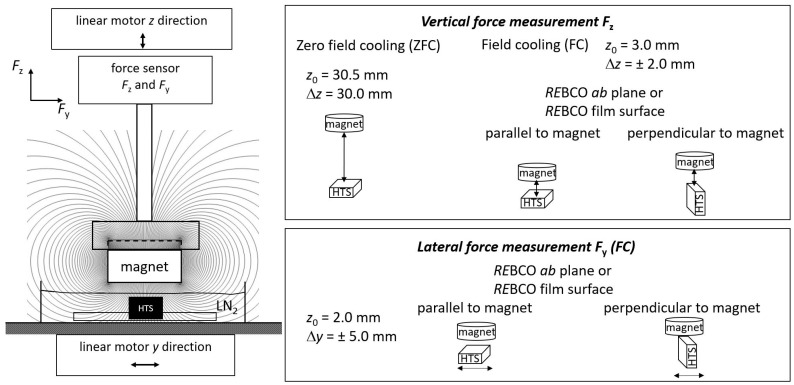
Schematic setup of the force measurement (**left**). The (**right**) panel shows the measurement procedure for vertical force measurement in zero field cooling (ZFC) and field cooling (FC) modes as well as for the different lateral force measurements.

Secondly, the force interaction between a permanent magnet and the superconductor was determined in different configurations. Figure 2 shows the schematic setup for these force measurements. The HTS samples were fixed to the setup and immersed in an open liquid nitrogen bath. This setup was connected to a linear motor, which allowed movement in the lateral *y* direction. On the opposite side, a cylindrical SmCo permanent magnet (Ø = 25 mm) was placed with an axial magnetization and a surface remanence *B*_surf_ = 0.38 T. The magnet was attached to a six-axis force/torque sensor (ATI Industrial Automation Inc., Apex, NC, USA) to determine the vertical force *F*_z_ and lateral force *F*_y_. This sensor array was connected to a linear motor enabling a movement in the vertical *z* direction. The reproducibility of the force values determined by several repetitions of the measurement is typically below 0.1 N; therefore, we assume a maximal error of about ±0.1 N for our data.

The vertical force *F*_z_ was measured in zero field cooling (ZFC) and field cooling (FC) modes as shown in Figure 2. For ZFC, the permanent magnet was positioned at a distance *z* = 30.5 mm above the superconductor, i.e., the magnetic field at the position of the superconductor can be neglected. Subsequently, the superconductor was cooled to 77 K with liquid nitrogen. Afterwards, the force *F*_z_ was measured while approaching the permanent magnet up to a minimum distance of 0.5 mm and subsequently withdrawing it to the original position, giving an overall position change of ∆*z* = 30 mm. In FC mode, the permanent magnet was positioned at a distance of *z* = 3 mm with respect to the superconductor and subsequently cooled down to 77 K. The vertical force *F*_z_ on the permanent magnet was measured using a distance change of ∆*z* = ±2 mm for two different sample orientations, i.e., with either the *RE*BCO *ab* plane (for bulks) or the HTS film surface (for tape stacks) parallel or perpendicular to the central magnetic field direction. It should be noted that the magnetic field of the permanent magnet in the superconducting area is predominantly aligned along the *z*-axis due to the significantly larger size of the magnet in comparison to the superconductor. In general, the vertical stiffness of SMBs can be determined from the ratio of the levitation force with respect to the displacement. From the FC mode measurements, the vertical stiffness *dF*/*dz* was determined from the slope of the linear fit for two complete hysteresis loops near the cooling position (i.e., close to *F*_z_ = 0). The mean value of the four slopes in the hysteresis loops was determined and is shown in the corresponding diagram.

Finally, the lateral force *F*_y_ was measured in FC mode, where the superconductor was cooled to 77 K at a distance *z* = 2 mm towards the permanent magnet. This force was recorded for a lateral movement of the superconductor of ±5 mm. Again, the lateral forces were measured for two different sample orientations, i.e., either with the *RE*BCO *ab* plane (for bulks) or the HTS film surface (for tape stacks) parallel or perpendicular to the central magnetic field. For the second case the superconducting samples were aligned in such a way that the *y*-movement was perpendicular to the *RE*BCO *ab* plane or the HTS film surface. The lateral stiffness *dF*/*dy* was determined from the slope in the linear fit of two complete hysteresis loops at a lateral displacement of ∆*z* = ±2 mm.

## 3. Results and Discussion

### 3.1. Trapped Field Measurements

**Figure 3:**
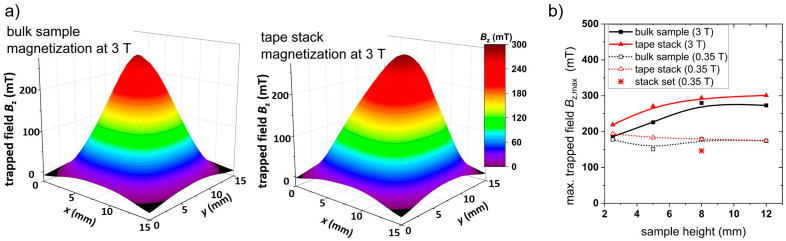
(**a**) Trapped field on the surface of the bulk and tape stack with a sample height of 8 mm; (**b**) Dependence of the maximum trapped field on the sample height. All data were measured at 77 K after magnetization at 3 T (closed symbols) and 0.35 T (open symbols). The lines are a visual guide.

At first, trapped field measurements were performed to compare the performance of the different samples. Figure 3a shows the trapped field in the *z* direction measured on the surface of the 8 mm thick bulk sample and a tape stack of the same height after magnetization at 3 T. The conical profile correlates well with the expectation from the Bean model [18]. The maximum trapped field of the bulk sample is *B*_z,max_ = 280 mT with the highest value in the center of the sample. The tape stack with the same geometry shows a maximum trapped field *B*_z,max_ = 294 mT. However, the cone exhibits a slight anisotropy as the peak is shifted towards one edge of the sample. This behavior results from the specific layer architecture of the coated conductor produced by Theva, where the c-axis of the *RE*BCO crystal structure is tilted by about 24° due to the texturing process of the MgO buffer layer resulting in an additional in-plane anisotropy of the critical currents [24]. Figure 3b summarizes the maximum trapped field *B*_z_ of the bulk and tape stack samples as a function of the sample height. The maximum trapped field of the bulk samples increases with the increase in sample height from *B*_z,max_ = 170 mT for a height of 2.5 mm to *B*_z,max_ = 280 mT for a sample height of 8 mm. A further increase in thickness does not result in a higher trapped field as the maximal gradient (which is equivalent to the maximal *J*_c_) is already achieved for the surface area of (12 × 12) mm^2^. A similar dependence on thickness was observed for bulk samples in previous studies [25,26]. The maximum trapped field in the tape stacks shows a similar dependence on the sample height with slightly higher values compared to the bulk sample, which might be explained with a higher critical current density of the YBCO film of the coated conductor. Again, a saturation of the maximum trapped field is observed for a sample height of about 8 mm, which is comparable to the results of other groups [27]. As the field gradient, which is directly proportional to the critical current density *J*_c_ according to the Bean model, reaches its maximum for a sample height between 5 and 8 mm, it can be assumed that *J*_c_ is saturated at this sample height. Magnetization of the superconducting samples with a NdFeB magnet at 0.35 T results in significantly lower trapped fields with maximum values *B*_z,max_ between 175 mT and 195 mT as shown in Figure 3b (open symbols). It can be concluded that the field trapping potential is not exhausted for both bulk samples and tape stacks with a thickness above 2.5 mm if a standard magnet is used for field cooling at 77 K. Therefore, magnet arrangements as flux collector setups or Halbach arrays [15] showing a higher surface remanence might be beneficial for such purposes.

### 3.2. Vertical Levitation Force Measurements

**Figure 4:**
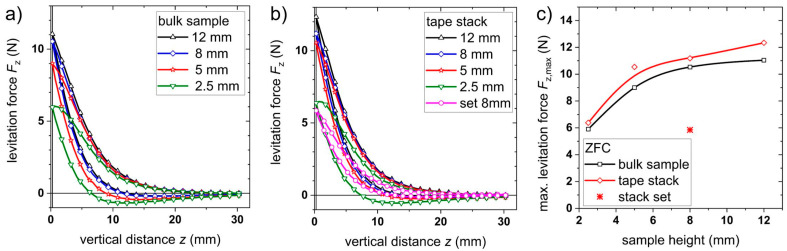
Vertical levitation force *F*_z_ at 77 K in ZFC mode for: (**a**) bulk samples and (**b**) tape stacks with different heights; (**c**) maximum levitation force *F*_z_ dependent on the sample height. The lines are a visual guide.

In the next step, levitation forces were measured as described in the experimental section. In ZFC mode, the maximal repulsive forces *F*_z_ in the vertical direction were determined as values between 6 N and 11 N for bulks depending on the sample height as shown in Figure 4a. In comparison, the maximum levitation force is between 6.4 N and 12.3 N for tape stacks; see Figure 4b. The maximum levitation force in the ZFC mode gives an indication for the maximum load applicable to a bearing. The observed thickness dependence of the levitation force is similar to the results of other groups measured either on bulk samples [26] or tape stacks [19,21,27,28]; however, the absolute values differ as they are dependent on the geometrical parameter of the bearing (i.e., size of the superconductor, magnet strength, etc.). According to the results in Figure 4c, the values are slightly higher for tape stacks with respect to bulks. At the same time, the hysteresis of the levitation force *F*_z_ is smaller for tape stacks if compared to bulk samples. In both cases, the hysteresis is larger for the thinner samples, which can be explained with the incorporation with more flux lines. As these flux lines are pinned in the material, they create an attractive force while retracting the sample from the minimum position (see discussion in [29]). In general, such hysteresis originates from a rearrangement of flux lines in the sample when the external magnetic field changes during the approach of the magnet to the superconductor. As a result, smaller hysteresis will lead to lower losses for movement along the *z* direction due to a reduced flux line movement.

**Figure 5:**
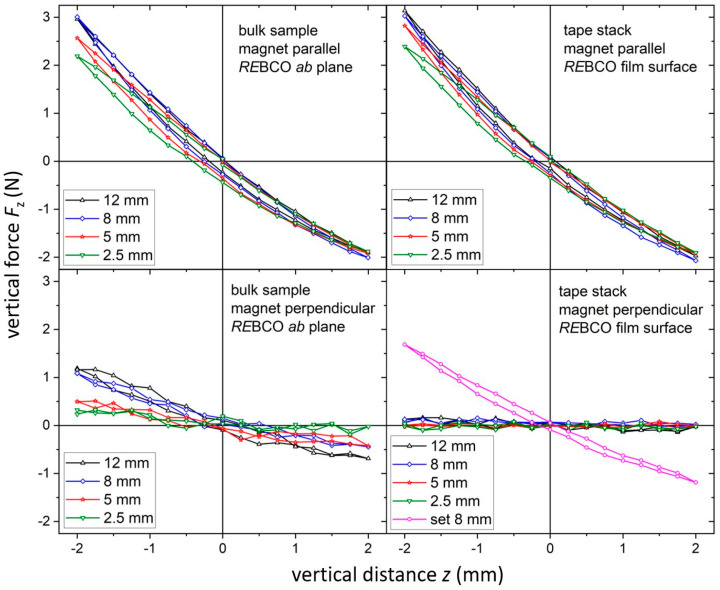
Levitation forces for a vertical displacement ∆*z* = ±2 mm after FC at a distance *z*_0_ = 3 mm between permanent magnet and superconductor for bulk samples and tape stacks in parallel (**upper row**) or perpendicular (**lower row**) sample position. The values were measured at a temperature of 77 K.

While the ZFC mode gives the maximum levitation force for an SMB setup, it is not suitable for most applications as the lateral restoring forces are small, resulting in an unstable levitation state. In contrast, the FC mode leads to stable levitation in hard superconductors due to an efficient flux pinning. As described above, the levitation force was determined for a vertical displacement of ±2 mm after FC at a distance of *z*_0_ = 3 mm between the permanent magnet and the superconducting samples. The results are summarized in Figure 5. Depending on sample height, the repelling force at maximum displacement reaches values between 2 N and 3 N, whereas an attracting force of about -2 N was determined for a magnet orientation parallel to the *RE*BCO surface. It is apparent that the vertical levitation force is equal or even slightly higher for tape stacks if compared to bulk samples of the same geometry. The differences in the hysteresis seem to be negligible. For a second measurement, the superconducting samples were rotated by 90° so that either the *RE*BCO *ab* plane (for bulks) or the *RE*BCO tape surface (for tape stacks) was perpendicular to the magnet surface. This allows the study of the anisotropy of the material in more detail, and the results are shown in the lower row of Figure 5. In this case, the measured forces are significantly reduced for bulks and almost negligible for tape stacks.

**Figure 6:**
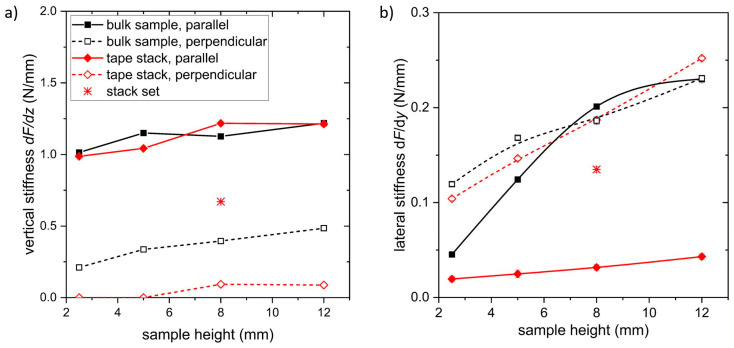
(**a**) Stiffness *dF*/*dz* for a vertical displacement ∆*z* = ±2 mm and (**b**) lateral stiffness *dF*/*dy* at a lateral displacement of ∆*z* = ±2.0 mm dependent on the sample height for bulk samples and tape stacks. The lines are a visual guide.

From the force data, the vertical stiffness *dF_z_*/*d_z_* was determined by the slope of curve around the cooling position (i.e., *z*_0_ = 3 mm). The results are shown in Figure 6a as a function of the sample height. If the *RE*BCO surface is parallel to the magnet, the vertical stiffness increases slightly with the increase in sample height, showing a saturation above a value of 8 mm for both bulk samples and tape stacks. A similar behavior was observed by other groups on bulk samples [30], whereas no data were available for tape stacks. This indicates that the magnetic field of the used permanent magnet is not strong enough to completely magnetize the superconductors during FC (see, for example, the magnetic field simulation in [31]). If the sample is higher than 8 mm, only the part close to the magnet contributes to the pinning of flux lines, whereas the other part has only a minor influence on the total levitation force.

For the second case with the *RE*BCO surface perpendicular to the magnet, the vertical stiffness is significantly reduced in bulks and tape stacks. The behavior is the result of several contributions. On the one hand, the surface area close to the magnet is reduced for thinner bulks or tape stacks due to the 90° rotation of the sample. This might explain the stronger thickness dependence of the stiffness for the bulk samples. On the other hand, the anisotropy of the material itself plays an additional role. For the bulk samples, the shielding current is no longer in the *ab* plane but needs to flow also along the *c* direction, where typically reduced values are observed. Additionally, the structural quality of the *RE*BCO bulks itself is deteriorated with the increase in distance from the surface due to the crystal growth process. Therefore, the lower parts of the bulk will carry smaller currents, which have a more severe influence if the bulk is rotated by 90°.

In the case of tape stacks, the reduced surface area as well as the intrinsic anisotropy of the superconductor itself plays a similar role to that of the bulk samples, whereas the influence of thickness on the structural quality of *RE*BCO can be neglected. However, the layered structure of the stack, in which the *RE*BCO layers are separated from each other by a significantly thicker substrate, needs to be considered as already discussed in the introduction. As a result, the shielding current can only flow within the separated superconducting layers, i.e., only in small cross sections, when the tape stack is rotated by 90° around the in-plane direction. This fact explains the almost negligible vertical stiffness for this orientation, i.e., the levitating system is not stable against forces along that direction.

### 3.3. Lateral Levitation Force Measurements

**Figure 7:**
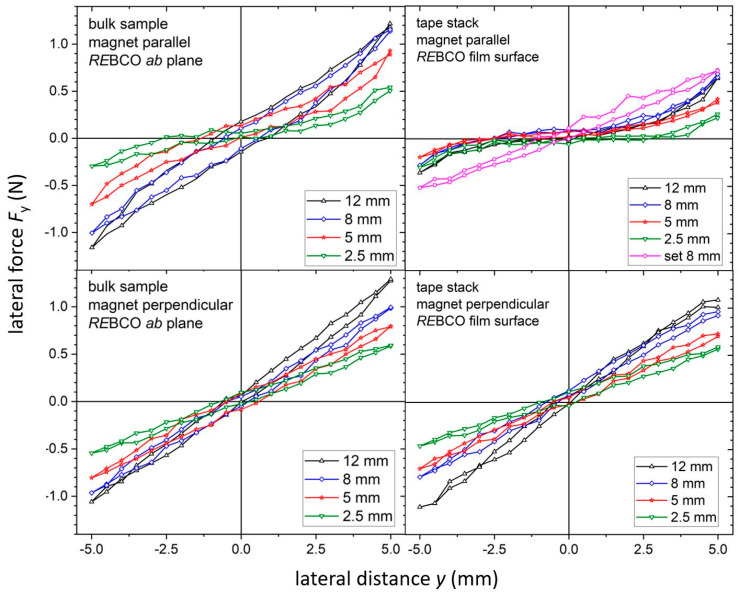
Lateral forces for a lateral displacement ∆*z* = ± 5 mm after FC at a distance *z*_0_ = 2 mm between a permanent magnet and the superconductor for bulk samples and tape stacks in parallel (**upper row**) or perpendicular (**lower row**) sample position. The values were measured at a temperature of 77 K.

In general, the lateral restoring forces *F*_y_, which act against a lateral displacement of the superconductor with regard to the permanent magnet, have to be considered in SMB setups as well. A high stiffness is required for such displacements in order to ensure a high stability of the bearing. The results of the lateral force measurements are summarized in Figure 7. In the case of bulk samples, the lateral forces reveal a hysteretic behavior when measuring the sample with their *RE*BCO *ab* plane parallel to the magnet surface as shown in Figure 7, upper row. The lateral force at a displacement of ±5 mm is about ±1 N for the bulk sample with a height of 8 mm. Similar measurements for tape stacks with the HTS surface parallel to the magnet surface reveal smaller values of about −0.3 N and 0.6 N for the 8 mm thick sample using a similar displacement. Again, all superconducting samples were rotated by 90° to align either the *RE*BCO *ab* plane (for bulks) or the *RE*BCO film surface (for tape stacks) perpendicular to the magnet surface. In this arrangement, lateral forces of about ±1 N were measured for the 8 mm thick bulk sample; see Figure 7, lower row. The major difference from the parallel arrangement is the significantly smaller hysteresis, which can be explained with a stronger pinning of the flux lines in the *RE*BCO *ab* plane laying parallel to the magnetic field in this case. The measurements on the tape stacks with the same alignment reveal almost similar lateral forces of about ±0.9 N for the 8 mm thick sample. Again, the hysteresis is quite small for this configuration.

The lateral stiffness was calculated from the levitation force measurements at a lateral displacement of ∆*z* = ±2 mm, as shown in Figure 6b. For this lateral displacement, the force is almost linear with a constant slope. Again, a strong dependence of the stiffness on the sample height was observed, which is in agreement to the results of other groups on bulk samples [30] and tape stacks [32]. Whereas a lateral stiffness of about 0.21 Nmm^−1^ was determined for the 8 mm bulk sample with the *RE*BCO *ab* plane parallel to the magnet surface, the value for the 8 mm high tape stack oriented in the same way is 0.03 Nmm^−1^, which is very low. This indicates an almost negligible lateral stiffness *dF_y_*/*d_y_* for tape stacks, i.e., the levitating system is not stable against forces from lateral direction. The difference between the bulk samples and the tape stacks arises from the local distribution of the superconducting material. Tape stacks are assembled from a number of *RE*BCO layers, which are separated from each other by a significantly thicker substrate. Therefore, superconducting currents can mainly flow in the film plane but are restricted to the film thickness perpendicular to the surface. At the same time, the magnetic flux lines oriented perpendicular to the film plane are split up in several segments due to the large distance between the neighboring *RE*BCO layers. These segments can be moved much more easily compared to the bulk material. In the perpendicular arrangement, the lateral stiffness was calculated to 0.19 Nmm^−1^ for the 8 mm high tape stack and bulk sample as well. Here, again, the flux lines are oriented parallel to the film plane, which results in a much stronger pinning strength.

### 3.4. Discussion

In general, these results indicate that the orientation of the superconducting layers with regard to the magnetic field is crucial for the levitation properties of the tape stack in any SMB configuration. In particular, the anisotropy of the levitation properties is extremely pronounced for tape stacks as the superconducting current flow in the *RE*BCO layers, which are separated from each other by the normal conducting substrates and the non-conducting buffer layers. Consequently, the lateral stiffness of a tape stack is almost zero if the *RE*BCO *ab* planes are oriented perpendicular to the magnetic field. The same applies to the vertical stiffness of tape stacks if the current-carrying *RE*BCO *ab* planes are oriented perpendicular to the magnet surface. Two solutions might be used to overcome this obstacle. On the one hand, the magnetic field profile might be adjusted in such a way that the flux lines are oriented parallel as well as perpendicular to the superconducting layers with a similar probability, giving rise to a sufficient vertical and lateral stiffness. Alternatively, a combination of stacks with different orientations to the magnetic field might be used for stable levitation in the vertical as well as in the lateral direction.

To test the second approach, a combined stack with a base area of (12 × 12) mm^2^ and a height of 8 mm was built as shown in Figure 1c. Similar measurements to those of the other samples were performed for this tape stack set. The results are included in the corresponding diagrams; see Figure 3, Figure 4, Figure 5, Figure 6 and Figure 7. The maximum trapped field of the 8 mm high stack set after field cooling with the NdFeB magnet was *B*_z,max_ = 146 mT, which is only slightly smaller than for the original tape stack. Measurements in ZFC mode show a maximum levitation force of 5.9 N in the vertical direction, which is only half of the full tape stack. A repelling force of 1.7 N and an attracting force of 1.2 N were measured for vertical displacement in FC mode, whereas lateral forces of about ± 0.6 N were determined. This results in a vertical stiffness of 0.7 Nmm^−1^ and a lateral stiffness of 0.13 Nmm^−1^ for the stack set. Table 1 gives an overview of the measured properties of the stack set in comparison to the bulk sample and the tape stack with the same height of 8 mm.

A stable levitation state in both the vertical and lateral directions can be realized by using a combination of stacks with different orientations to the magnetic field. However, the maximum load in ZFC mode is reduced by almost 50%. This reduced levitation force might arise from the fact that only the middle part of the stack set with an area of (12 × 8) mm^2^ contributes to levitation in this case. In addition, this area might be even reduced due to disturbed regions close to the cut edges, which can have a width of up to 100 µm as revealed in SEM investigation. Further optimization steps are required to adjust the area of the differently oriented tape stacks to the used magnetic field profile in the bearing to achieve the best levitation performance.

## 4. Conclusions

Pieces of superconducting REBCO tapes, which were originally developed for current transport in cables, were arranged as tape stacks for detailed study and might replace the typically used bulks in SMBs. These tapes are characterized by a significantly higher critical current density if compared to the bulk material. Therefore, high trapped fields and levitation forces are expected with an effective flux pinning inside the superconductor. To study the behavior of such tape stacks in detail, stacks with different height were prepared and compared to bulk samples with the same size. In particular, the influence of the sample height on properties such as trapped field and levitation forces was investigated. In all cases, improved properties such as trapped fields, levitation forces or stiffnesses in the vertical and lateral directions were measured with the increase in sample height up to a thickness of about 8 mm. A further increase in the bulk or stack height did not lead to any further improvement of the properties for the configurations studied here. For tape stacks, a strong anisotropy of the levitation properties was found. In the vertical direction, the levitation forces of tape stacks are even slightly higher than for bulk samples of the same geometry when the superconducting layers are aligned perpendicular to the magnetic field direction. However, the lateral forces are almost absent for this configuration. The opposite behavior was observed when the tape stack was rotated by 90° around the in-plane direction. Consequently, it is important to arrange the tape stacks as a set with their superconducting layer parallel and perpendicular to the magnetic field in rotationally symmetric SMBs to ensure high stability in all directions.

In general, there are different ways to realize an optimized configuration. On the one hand, one can use a combination of tape stacks with vertical and lateral alignment as it was tested in this work. This results in a significantly improved lateral stiffness; however, this sacrifices some of the vertical stability. Future studies need to determine an optimized volume ratio between the two stack orientations for a given magnetic field geometry. On the other hand, the magnetic field distribution might be adjusted with regard to the stack alignment. As already mentioned, the magnetic field of the studied setup was predominantly aligned along the *z*-axis due to the significantly larger size of our axially magnetized magnet in comparison to the superconducting samples. Instead, one might use magnet configuration as flux collector arrangements or Halbach arrays with strong magnetic field gradients in the superconducting area. In this case, finite element simulations might be particularly helpful in finding the best configuration for a given SMB geometry.

## Figures and Tables

**Table 1:** Comparison of the trapped field after magnetization at 0.35 T, the maximum force in ZFC as well as the vertical and lateral stiffness for different orientations for the bulk sample, the tape stack and the stack set with a height of 8 mm.

	Trapped Field *B*_z max_ (mT) (Magnetization at 0.35 T)	ZFC *F*_z max_ (N)	Vertical Stiffness *dF*/*dz* (N mm^−1^)		Lateral Stiffness *dF*/*dy* (N mm^−1)^	
			ǁ	_┴_	ǁ	_┴_
Bulk sample	179	10.5	1.1	0.4	0.21	0.19
Tape stack	179	11.2	1.2	0.1	0.03	0.19
Stack set	146	5.9	0.7		0.13	

## Data Availability

All data in this work are available on request by contact with the corresponding author.
